# Respect, trust and continuity: A qualitative study exploring service users’ experience of involvement at a Healthy Life Centre in Norway

**DOI:** 10.1111/hex.12846

**Published:** 2018-11-24

**Authors:** Espen Sagsveen, Marit B. Rise, Kjersti Grønning, Heidi Westerlund, Ola Bratås

**Affiliations:** ^1^ Department of Public Health and Nursing Faculty of Medicine and Health Sciences Norwegian University of Science and Technology Trondheim Norway; ^2^ Department of Mental Health Faculty of Medicine and Health Sciences NTNU Trondheim Norway; ^3^ KBT Mid‐Norway (Resource Centre for Service User Experience and Service Development) Trondheim Norway

**Keywords:** health behaviour, healthy lifestyle, primary health care, qualitative research, user involvement

## Abstract

**Background:**

To meet the challenges caused by non‐communicable diseases, Norway has established Healthy Life Centres within primary care to encourage a healthy lifestyle. To promote people's health and ensure high‐quality services, user involvement in contemporary health care is regarded as essential.

**Objective:**

To explore the experience of user involvement among Healthy Life Centre users participating in individual health consultations, followed by physical activity groups and/or diet courses.

**Methods:**

This was a qualitative study based on twenty semi‐structured individual interviews conducted between September 2015 and May 2016 at a Healthy Life Centre in Norway. Data were analysed using systematic text condensation.

**Results:**

Being respected and having a trustworthy relationship with the professionals were found to be essential for the service users’ involvement. Building a trustworthy relationship was disrupted for some service users by a lack of relational continuity. This lack of continuity jeopardized the continuation of professionals’ awareness of the service users’ challenges and personal goals. The service users’ preferred levels of user involvement varied. Some service users did not always want to play an active part and instead wanted the professionals, as “experts,” to decide.

**Conclusions:**

The findings imply that the professionals need to assess each service user's desires for involvement and consider how these can be met. Thus, user involvement cannot be understood without considering the particular setting and each individual service user's preferences for involvement. Relational continuity is needed to maintain the service users’ challenges and goals throughout the services and to promote health behaviour changes.

## 1 INTRODUCTION

The rise of non‐communicable diseases (NCDs)^1^, ^2^, ^3^, ^4^ is primarily caused by a few common and preventable behavioural risk factors, such as physical inactivity, an unhealthy diet, tobacco use, overweight and obesity and harmful alcohol consumption.^2^, ^3^ To meet the challenges caused by these lifestyle‐related risk factors, Norway has established Healthy Life Centres (HLCs) within primary care as a part of a national strategy to promote a healthy lifestyle and prevent NCDs. The HLCs offer knowledge‐based support for health behaviour change through individual and group‐based counselling and activities.^5^, ^6^, ^7^

To promote people's health and ensure high‐quality services, user involvement is regarded as essential in contemporary health care.^8^, ^9^, ^10^, ^11^, ^12^, ^13^, ^14^ User involvement has potential benefits such as supporting health behaviour changes, empowering people to take more responsibility, increasing patient satisfaction and adherence to treatment, as well as safety and quality of care.^13^, ^15^, ^16^ Through policy initiatives, service users are encouraged to act as active participants in their own care and decision making, expressing their individual needs.^17^, ^18^, ^19^, ^20^, ^21^, ^22^ In line with this, policy initiatives and regulations encourage and instruct health‐care professionals to provide a service promoting user involvement, both on an individual and system level.^18^, ^23^, ^24^, ^25^ To ensure the service users′ right to be involved as well as the health professionals’ duty to do so, several countries such as England and Norway have legislated user involvement.^26^, ^27^, ^28^, ^29^, ^30^

Previous studies have found that service users are motivated to participate and that they find participation important and valuable.^31^, ^32^, ^33^ It is worthwhile to consult service users about their opinions and to share and receive information about rehabilitation, rationale for treatment, progress and recovery.^31^, ^32^, ^33^ Service users emphasize collaboration and a mutually respectful and equal relationship as fundamental in user involvement.^18^, ^33^, ^34^, ^35^ Dialogue and knowledge sharing were found to be essential to empower the service users to participate and achieve shared decision making.^18^, ^33^, ^34^, ^35^ Barriers towards participation are lack of health professionals’ skills, empathy and adequate attitude to share knowledge, power and responsibility.^18^, ^32^, ^35^ Being met with a paternalistic attitude and not being acknowledged as competent and equal partners in decision‐making situations can create a feeling of being omitted from involvement.^18^, ^35^, ^36^ However, studies show that the service users’ desired level of involvement is influenced by the stage and severity of illness.^37^, ^38^, ^39^ Other studies have found considerable variation in patients’ level of preferred participation and a mismatch between levels of preferred and received participation.^14^, ^31^, ^32^

The HLCs guidelines state that service users should be actively involved in the planning, delivery and evaluation of services.^26^, ^28^ According to the guidelines, user involvement includes each service user's rights and possibilities to have a genuine influence on his or her service provisions.^7^

Because involvement of the service users is considered as essential in health promotion initiatives that encourage health behaviour change and aim to enable people to increase control over their health,^8^, ^40^ it is important to address the service users’ own experiences of involvement.

Accordingly, there is a need to explore the service users’ perspectives on what may facilitate and hinder active involvement. Such knowledge is necessary to illuminate the essential knowledge, skills and attitudes to practice user involvement in line with the service users’ preferences and values.

Hence, the overall aim of this study was to explore the experience of user involvement among HLC service users participating in individual health consultations, followed by physical activity groups and/or diet courses. More specifically, we aimed at exploring what facilitated or hindered their involvement at the HLC and how involvement might contribute to changing health behaviours related to physical activity and healthy eating habits.

## 2 METHODS

This was a qualitative study based on semi‐structured face‐to‐face individual interviews with service users in an HLC in Mid‐Norway. Individual interviews were chosen as an appropriate method of data collection to obtain in‐depth insight into the service users’ experiences and perceptions.^41^, ^42^ The interviews were conducted between September 2015 and May 2016.

### 2.1 Study setting

The HLC is an easily accessible primary care service that people can attend through referral from a general practitioner, other health‐care provider or by self‐referral.^7^ The HLCs direct their services towards people who need help to change living habits related to healthy diet, physical activity and tobacco cessation.^7^, ^43^ Intervention periods of 12 weeks are offered, with the possibility to extend the intervention twice; hence, at some HLCs, a total of 36 weeks of intervention can be experienced. The intervention period usually starts and ends with an individual health consultation lasting approximately 60 min. The HLC in this study offered physical activity groups, both in‐ and outdoors, two times a week lead by a physiotherapist. Between fifteen to twenty service users are usually present at the training sessions. The training sessions included elements of circuit‐based training with endurance and resistance exercises combined with coping and joy through exercise. The centre also offered healthy diet courses composed of five sessions of two hours each lead by a clinical dietitian. Approximately ten service users were included in each course. The course provided practical and theoretical information necessary for developing and maintaining good and healthy eating habits. This included information about food and eating recommendations, nutrients, product declarations and cooking classes.

### 2.2 Recruitment

HLC service users of both genders, representing a variation in age and duration of participation at the HLC, were recruited. Inclusion criteria encompassed adults with experiences from a 12‐week intervention period with individual health counselling and participation in a physical training group and/or dietary course. The first author recruited service users by introducing the study in three training groups and three dietary courses. Information about the study was given both orally and written through an information sheet. Two service users were not included because they did not have any experiences with the individual health counselling. All together approximately seventy‐five service users were potential participants in the study. The service users were accepted consecutively. All service users signed an informed written consent form before taking part in interviews.

### 2.3 Data collection

The interviews were conducted by the first author at a location chosen by the service users. One interview was conducted in the participant's home—the others were in a meeting room at the locations housing the HLC. The interview duration ranged from 22 to 87 min with an average of 54 min.

All interviews were conducted according to an interview guide. To develop the interview guide, the first author conducted a literature review and discussions with co‐authors; one co‐author had extensive research experience on user involvement. The interviews were initiated using open‐ended questions inviting the service users to talk about their own experiences, encouraging them to illustrate their experiences with examples. The main questions are displayed in Table 1. The questions were used as a guide, and the sequence was dependent on the service users’ response to previous questions. All interviews were audio‐recorded and transcribed verbatim by the first author and a professional transcriptions service. The study was approved by the Norwegian Data Protection Official for Research (NSD) (Project no. 43803).

**Table 1:** Main topics in the interview guide

Could you describe for me how the initial health consultation was conducted and the content of this? ○ How were you involved in the goal setting? ○ How was the activity tailored to your goals?
How would you describe your influence on the content and delivery of the diet course or training activity? ○ To what extent do you want to be involved in planning and delivery? ○ What is your motivation to exert influence? ○ What significance does it have for you to be involved? ○ How do you experience that it is facilitated for you to be involved?
When I say service user involvement, what does it mean to you?

### 2.4 Data analysis

After each interview, the first author listened through the digital audio recording and wrote a summary. After 20 interviews, no new or relevant data emerged, and the information gathered was found sufficiently saturated for analysis.^44^

Analysis was conducted as collaborative negotiations between the five authors, who represent various backgrounds and research experiences, such as nursing, psychology and entrepreneurship and innovation. One of the authors has extensive experience as a service user and works in a resource centre for service user experience and service development.

Systematic text condensation (STC) was used because it offers a process of intersubjectivity, reflexivity and feasibility during data analysing and is a structured and well‐described systematic method for analysing qualitative data.^44^ Further, STC focuses on thematic analysis of meaning and content of data across cases and was thereby useful for our study. The STC procedure consists of four steps.^44^ First, all transcripts were read by all five authors to establish an overview and to gain a general impression of the data, searching for preliminary themes related to the service users experience of user involvement. At this stage, bracketing preconceptions, attempting to mitigate the deleterious effects of unacknowledged preconceptions, was important.^45^ After reading the transcripts, all authors met to discuss the preliminary themes found. Examples of preliminary themes were “goal setting and ownership to change,” “adjustments and flexibility,” “involvement in standardized services” and “relationship and trust,” Next, the first author systematically reviewed the transcript to identify meaning units representing different aspect of the service users’ experience with user involvement. Third, the first author classified and sorted the meaning units into code groups, followed by a common agreement between the authors about the content of the codes. Further, the first author systematically abstracted meaning units within each of the code groups and then sorted them into subgroups. Then, a condensate was abstracted from each subgroup, merging the content from the meaning units of this subgroup. Examples of code groups and subgroups were (a) “personal goal setting” with the subgroups “to feel ownership”; and “feeling responsibility”; and (b) “respect and trust” with the subgroup “to be seen.” After finishing the condensation, illustrative quotations were identified. Finally, the condensed contents were synthesized to generate generalized descriptions and concepts (recontextualized) concerning service users’ experiences with user involvement at the HLCs. These are described as the final themes in the presentation of the results. The research group validated the interpretations and findings against the initial transcripts to ensure that the synthesized result reflected the original context.

During the analysis process, preliminary results were presented and discussed with a research group in patient education and patient involvement where four of the authors are members. Preliminary results were also presented and discussed at a national HLC conference and at a national seminar with service user representatives. The discussions helped to validate the analyses and interpretations of the findings.

Quotes from the transcripts were translated into English by the first author (ES) and then cross‐checked by the other authors to verify the meaning of the content. Quotes are used in the results presentation to elaborate and illustrate the findings and entail information about the service users’ gender, age, participation in exercise group and/or diet course and a participation code.

## 3 RESULTS

Twenty service users from a HLC located in Mid‐Norway participated in individual semi‐structured interviews. The sample consisted of 20 HLC service users, 16 females and four males with a mean age of 52 years (range: 24‐73). Nine service users had finished or were in their first 12‐week intervention period, seven had finished or were in their second period and three were in their third period. Sample details are shown in Table 2.

**Table 2:** Demographic characteristics of the study sample (N = 20)

Characteristics	Number of participants
Gender	
Female	16
Male	4
Age (mean 52.6, range 24‐73)	
24‐41 years	4
42‐55 years	7
56‐73 years	9
Number of 12‐week periods	
One	9
Two	8
Three	3
Referral from	
GP	11
Hospital clinics	4
Psychologist	2
Self‐referral	3
Kind of service attended	
Only exercise group (E)	5
Only dietary course (D)	4
Both exercise and dietary	11

Analyses of how the service users experienced user involvement in the HLC resulted in four main themes: (a) having a trustworthy relationship; (b) feeling ownership and responsibility through personal goal setting; (c) trusting the professionals’ decisions; and (d) service users’ experiences of involvement in group activities.

### 3.1 Having a trustworthy relationship

The service users said that feeling respected, acknowledged and having a trustful relationship were essential parts of being involved in their lifestyle change process, both in the individual health consultations and in the group activities. They described that they were taken seriously and met with respect when they told the HLC professionals about their health challenges during the individual health consultations. This was described as a prerequisite for being open about their own health challenges and getting involved in the process to change their health behaviour. The service users conveyed that this was due to the professionals showing a genuine interest in them and their challenges.

I felt that I was taken seriously when telling them about my challenges, and being open about my challenges were met with great respect. This was very important for me if I should try to start the process here at all. (30‐year old female, exercise and diet, P9)

Not all of the service users had met the same professional in the individual health consultations and the group activities. Those who did describe it as positive since the professional then had first‐hand knowledge about their health challenges and personal goals. Having the same professional in both settings was described as making the participant feel safer and made it easier to give feedback to the professional. To experience follow‐up by the same professional in both settings was also reported to make it easier for the service users to relate the skills and knowledge achieved at the HLC to their own life situation and change in health behaviour.

I feel good because she is fully updated on what I am struggling with, compared with another instructor that I might have had to inform again (….) I feel safe since she knows about my problems. I think she understands that I have some limitations and challenges, and because of that I feel safe. (59‐year old female, exercise and diet, P15)

Those who met different professionals in the health consultations and group activities said it could be more difficult for the professional to see and understand everyone's challenges because they did not know their personal goals. One of the service users said that she would prefer a meeting with both professionals in her transition between the individual and group sessions.

My impression was that the training instructor did not know my goals. Not until we, after a while, had a meeting, me and the two instructors from the HLC. There we talked about my goals and she did not seem to know my goals or my processes. So, in a way it seemed like she was there primarily for the training. (30‐year old female, exercise and diet, P9)

### 3.2 Feeling ownership and responsibility through personal goal setting and support

When the service users described how they were involved in their lifestyle change process, they emphasized the individual health consultations and defining personal goals.

But I would like to say that the way we are followed up in the individual consultations here are good examples of user involvement. So, in the consultations I feel, at least for my part, that I, as a service user, am being put in the centre. (30‐year old female, exercise and diet, P9)

The service users further discussed the goals as their own and underlined that they were responsible for doing what was necessary to reach them and to maintain the lifestyle changes. They reported that only they, themselves, really knew what their challenges were, suggesting that it was negative if someone else decided for them what should be their goals. They emphasized that the process of making changes had to be theirs.

It is my life and my challenges. When someone else comes and tells me what my challenges are then I do not get any ownership to the process. (…) Because it is difficult to become aware of your own challenges when you cannot influence. (30‐year old female, exercise and diet, P9)

However, they said that without support from the HLC professionals, friends and family, they would not be able to make these changes. According to the service users, the HLC professionals helped them reflect upon their goals, guiding them in a careful and honest way towards goals that were specific and attainable. The service users expressed that they appreciated the HLC professionals guiding them in adjusting their goals.

I am not good at goal setting. It is for sure important and sensible, but I have had trouble figuring out what I want the resent years. And I have had some goals, but I have failed to reach them, and then I get disappointed. (49‐year old female, exercise, P2)

The service users also described how the relationship to the HLC professional and other service users made them feel obliged in a positive way to attend to the group activities. This was exemplified by describing how the professionals and other service users were waiting for them to show up in the group sessions, even calling them if they did not meet. As they mentioned, this made them feel responsible and committed to partake in the service they voluntary had signed up for.

For me it is about having someone who can push me in the right direction and someone to talk with when you need that. Having someone walking “the road” along with me. That is important for me. (49‐year old female, exercise, P2)

### 3.3 Trusting the professionals’ decisions

When talking about the services at the HLC, the service users said they were satisfied, giving positive descriptions of the professionals as confident, skilful, friendly, solution‐oriented and good at explanations and answering questions.

She was very good at asking elaborating questions and asking again if there was something she did not understand. She threw the ball with me in a way, asking questions like: “Yes, did I understand it right?”, “Will it work to do it like this?”. (30‐year old female, exercise and diet, P9)

The service users said they had confidence in the HLC professionals’ skills and experienced the service to be of high quality and built on research‐based knowledge. Almost all service users emphasized that they would participate in the HLC services as long as they could. Being satisfied with the HLC services was given as a reason why they had not thought about or felt any need for more involvement in the training sessions and dietary courses. While they described the importance of being involved in defining personal goals, they were less concerned about being involved in how to reach the goals and the planning of the activities. One participant said that involvement was “a waste of time” since “the training sessions and diet course already were of high quality.” The service users said they were inexperienced when it came to knowledge about training, but described the HLC staff as skilful and confident professionals. This was also described as the reason why they let the professionals decide and did not involve themselves in planning and organizing the activities. Some also said they preferred that someone else made the decisions due to their own psychological distress.

I think it is fine actually, at least where I am in my life right now, that someone else decides. That I do not need to make decisions about things or have to come up with proposals, because I am so unfocused. So, for me it is good that it is a fixed arrangement, or varied, but at the same time fixed, to put it that way. (49‐year old female, exercise, P2)

The service users’ wish for involvement also depended on how long they had attended the HLC service. They said they expected to be more involved the longer they attended the service, relating this to both their own and the HLCs professionals’ goal of being independent from the HLC service. However, the service users did not describe being directly involved in the planning and organization of the training sessions and dietary courses. When asked about the importance of being involved, all service users, except one, said that they had not reflected upon this.

I accept the service the municipality gives me. I do as I am told. Since I am satisfied, I do not really think much about how I can have an impact on my activities. We humans are strange; we follow the herd and do as we are told. (49‐year old female, exercise, P4)

When asked whether the service users experienced any clear expectations from the professionals to be actively involved in the planning and organization of the activities, the service users said that they did not. However, they described a feeling of openness to come forward with proposals and feedback.

Not everyone is so tough that they dare to speak out. I know that I'm not always that tough. It's about my situation here and now. So, it is perhaps a bit difficult to speak out like it is right now. That you do not feel that there is enough openness or a proposal box available. (49‐year old female, exercise, P2)

### 3.4 Service users’ experiences of involvement in group activities

While the service users described being involved through the individual health consultations, their experience of involvement in group activities was described as somewhat more limited. The service users said their involvement in the group activities took place through individual adjustments, dialogue and the possibility to ask questions related to their personal challenges. If there were exercises they did not manage to do, the instructor suggested alternatives.

They have their arrangement for the service provision. I can say what my goals are. If I cannot follow exactly what they have planned, they must come up with alternative training. So, that type of user involvement I see practised. I did not manage some exercises and when I told them, I got it adjusted. (49‐year old female, exercise, P4)

Other examples of involvement expressed were evident in selecting which days they wanted to exercise and whether they wanted to exercise in‐ or outdoors. In the dietary course, the service users said their involvement consisted of asking questions, talking about their challenges, sharing experiences and having further follow‐up by the HLC dietitian.

I think it was an opportunity in the course to participate and influence by asking questions and front your personal opinions, and the course leader challenged us with homework. (59‐year old female, exercise and diet, P15)

Despite positive experiences of individual adjustments and the professionals’ intentions of seeing everyone, some described barriers for involvement and adjustments in the group settings, due to standardized frames and limited resources.

We were many persons with very different challenges. It felt in a way like being lead through a standardized course where everyone in a way should fit in. So, I would like to, I do not really know how, have had some more influence there. I would really like to have that. (30‐year old female, exercise and diet, P9)

However, they did not expect the health professionals to adjust every training session to everybody's needs and wishes, as they knew they all had their personal luggage and various reasons for attending the activities.

Everyone has a luggage here, but she cannot go into every session and adjust it to everyone's needs. She has to carry out the training, but the exercises are maybe adjusted, unconscious, to everyone. (49‐year old female, exercise, P4)

## 4 DISCUSSION

Our findings showed that being respected, acknowledged and having a trustworthy relationship with the professionals were considered a prerequisite for the service users’ involvement in changing their health behaviour. This in line with other studies showing that user involvement is facilitated by a personal relationship, based on respect, collaboration and knowledge sharing.^16^, ^18^, ^36^, ^39^, ^40^ Similarly, Følling and colleagues found that HLC participants emphasized emotional support as essential to give them the courage to start and continue with healthy behaviour changes.^46^ Thus, our findings highlight the importance of the trained HLC professionals possessing effective communication skills, being emphatic and able to build trust, respect and partnership.

However, a trustworthy relationship was hampered for some service users by a lack of relational continuity, that is, meeting different professionals in their individual health consultations and group activities. A relationship of trust has been found to be built on relational continuity with the same professional, giving greater psychosocial security for the service users in consultations.^47^ Further, relational continuity enables the professional to gain specialized knowledge of the service user's condition and become familiar with the service user's consulting behaviour.^40^, ^47^, ^48^ Our findings suggest that the group size and meeting different professionals in consultations and group activities are structural barriers within the HLC service that may hinder relational continuity from the transmission between the individual health consultation and the group activities. This lack of continuity may jeopardize the professionals’ awareness of the service users’ challenges and personal goals. Hence, our findings highlight the importance of relational continuity by transferring knowledge and information from individual consultations into group activities. Thorough knowledge of the personal challenges and goals provides professionals with the possibility to motivate the service users by referring to their personal goals, thus facilitating intrinsic motivation for health behaviour change.^49^, ^50^ In addition, it provides the service user with an opportunity to reflect with the professional on how their experiences from the group activity can be used to promote lasting health behaviour change in everyday life. Følling and colleagues found that HLC participants desired long‐lasting change, avoiding temporary solutions that would ultimately fail.^46^ Hence, our findings indicate that the individual health conversations are an important means to support the service users’ health behaviour change.

Our results showed that the preferred levels of user involvement varied and that the service users had various preferences of involvement. This in line with other findings showing that role preferences vary considerably across service users^32^ and that not all clients prefer to participate at all times.^51^ The service users’ preferred level of involvement is found to be related to their role expectations.^14^, ^16^ Authors have argued that in the centre of user involvement resides a redefinition of the client role, where the historically “paternalistic” model has been transformed towards perceiving the service user as an active participant.^14^, ^24^ In contrast to the policy initiatives fronting an active service user role, our findings indicate that some service users prefer a more passive role where health professionals make decisions on their behalf. Our findings emphasize the importance of the health professionals assessing the service users’ role expectations and to what degree the service users want to be involved in decisions.

Our findings further showed that the service users’ feeling of being involved differed between the individual consultations and groups activities. The service users felt actively involved during goal setting in the health conversation. This in line with other findings showing that service users perceive being active participants in the phases of goal setting.^32^, ^52^ Being involved and making decisions about personal goals may have several benefits such as increased confidence and sense of ownership.^52^ In the present study, the service users expressed this by emphasizing that their goals had to be their own. Previous research also indicates that involvement in goal setting is an essential component of dietary behaviour change and increased physical activity, both to initiate and maintain change.^46^, ^50^, ^53^, ^54^

Simultaneously, some service users preferred that the professionals guided them towards realistic goals. They had not thought about or did not feel any need to be involved. Previous studies have shown that service users in some cases consider the professionals as the “experts” with specialist knowledge and thus better suited to set the goals and decide how to reach them.^32^, ^52^, ^55^, ^56^ The lack of knowledge and self‐confidence may also be a main barrier for involvement in the goal setting process.^14^, ^32^, ^52^ Although seeing the professional as the “expert” has been found to make service users feel disempowered,^52^ our findings did not reveal any feelings of disempowerment. Instead, service users described guidance by the professionals as positive, preferring a more passive role due to their own psychological distress and inexperience. This is in accordance with findings showing that psychological distress and disease can challenge user involvement^14^, ^16^, ^18^ and health behaviour changes.^43^, ^46^ The service users’ choice of letting the professionals decide has also been considered as result of a service user‐professional relationship characterized by trust, which gives confidence to allow others to decide on one's behalf.^51^ This highlights the importance of health professionals making distinctions between desired and achieved levels of involvement for each service user. The service users’ choice of letting the professionals decide should be considered an active choice and not a result of passivity or lack of involvement. This implies that the professionals need to assess each service user's wish and requirement for involvement and how to meet these.

### 4.1 Strengths and limitations

A strength in this study is that the analyses and writing of the paper were conducted by a group of researchers with various backgrounds. To include different perspectives, the research group consisted of persons with different professional backgrounds and practical experiences from nursing, health sciences, psychology and public and mental health. Notably, the fourth author (HW) is a public representative with extensive knowledge and experience working with user involvement. The contribution of different perspectives and the rigorous analytical process probably helped strengthen the reliability and accuracy of the findings.^57^ To strengthen the validity of the analysis, the interpretations and findings were cross‐checked against the initial complete transcript during the various steps of analysis.^44^ However, the possibility that the researchers’ preconceptions or attitudes affected the interpretation of data cannot be excluded. One step taken to avoid preconceptions affecting the interpretation was critically discussing the interpretations between the co‐authors with various backgrounds and in a research group of experienced researchers.

A possible limitation in this study is the self‐selection of the sample. Those who volunteered to take part in interviews may have been those who were particularly positive to the service provision and involvement, and this might have influenced the results. However, the service users varied regarding gender, age, experiences with the diet course and/or physical activity groups and number of weeks receiving HLC services. This study was conducted among HLC service users at a single HLC. This might limit the transferability of the results to other settings, especially since the HLCs differ significantly in organization and size. The HLC in this study was located in a big city, and all findings may not necessarily be transferable to small HLCs in rural municipalities. On the other hand, the findings are supported by research in other contexts, indicating that the findings can be transferable beyond the present context, thus strengthening the external validity.^58^

## 5 CONCLUSION AND IMPLICATION FOR CLINICAL PRACTICE

This study highlights the importance of trained HLC professionals possessing effective communication skills, showing empathy and being able to build trust, respect and partnership. However, building a trustworthy relationship was hampered for some service users by a lack of relational continuity that may hinder relational continuity and jeopardize the professionals’ awareness of the service users’ challenges and personal goals. Hence, further work at the HLCs should consider how relational continuity could be enhanced, such as continuity with the same HLC professional and transfer of information about each service user's goals and personal challenges throughout the HLC period to promote lasting health behaviour change. Our findings indicate that the individual health conversations and goal setting are an important mean to individualize the service and to promote health behaviour change in the service users’ everyday life setting. Further, our findings show that some service users do not always want to have an active part and instead prefer to let the professionals as “experts” decide. This underpins the importance of HLC professionals making distinctions between desired and achieved levels of involvement for each service user. The service users’ choice of letting the professionals decide should be considered an active choice and not merely because of passivity or lack of involvement. Hence, our findings imply that the HLC professionals need to assess each service user's desires and requirements for involvement and how these can be met. In that respect, user involvement cannot be understood without considering the particular setting and each individual service user's preferences for involvement.

Further research is needed, both qualitative and quantitative studies, investigating the service users’ and professionals’ perspectives and preferences for involvement in order to reach a broader perspective on factors that facilitate and hinder user involvement at HLCs. Future research should investigate how service users and professionals interact in relation to user involvement both in consultations and group activities through observational studies. In addition, studies exploring and investigating structural interventions in HLCs to strengthen a continuity of information about the service users’ goals and challenges should be conducted.

## CONFLICT OF INTEREST

The authors declare that they have no competing interests.
